# The obscurance of the greatest sylvatic yellow fever epidemic and the cooperation of the Pan American Health Organization during the COVID-19 pandemic

**DOI:** 10.1590/0037-8682-0787-2020

**Published:** 2020-12-11

**Authors:** Carlos Frederico Campelo de Albuquerque e Melo, Pedro Fernando da Costa Vasconcelos, Luiz Carlos Júnior Alcantara, Wildo Navegantes de Araujo

**Affiliations:** 1 Universidade de Brasília, Programa de Pós-Graduação em Saúde Coletiva, Brasília, DF, Brasil.; 2 Instituto Evandro Chagas, Ananindeua, PA, Brasil.; 3 Fundação Oswaldo Cruz, Instituto Oswaldo Cruz, Rio de Janeiro, RJ, Brasil.; 4 Universidade Federal de Minas Gerais, Programa de Pós-Graduação em Genética, Belo Horizonte, MG, Brasil.

**Keywords:** Yellow fever, Disease outbreak, Epidemic, Communicable disease control, International cooperation, Pan American Health Organization

## Abstract

**INTRODUCTION:**

Since 2016, Brazil has been in the midst of its largest sylvatic yellow fever epidemic ever, found predominantly outside the Amazon region. Cases originating from Brazil have been reported in France, the Netherlands, Romania, Switzerland, Argentina, and Chile. The epidemic began in the Central-West region of Brazil in 2014, spreading into the Southern region, with significant non-human primate transmission continuing towards Paraguay and Argentina.

**METHODS:**

This report is an integrative review of Pan American Health Organization cooperation during a sylvatic yellow fever epidemic.

**RESULTS:**

The Pan American Health Organization has played a central role in handling the yellow fever emergency, collaborating with the Ministry of Health and various research groups in supporting interventions of different response areas. The Pan American Health Organization's technical cooperation included: training and workshops to exchange experiences, carrying out technical cooperation in patient management and epidemiological, entomological, laboratory, and epizootic surveillance, organizing the assistance network, and acquiring strategic inputs. The Pan American Health Organization’s technical cooperation supported the Ministry of Health’s decision to adopt a single-dose vaccine and use fractional doses to support the vaccination needs of more than 39,000,000 people. The coronavirus disease 2019 pandemic contributed to the failure of reaching the yellow fever vaccination goals and made it difficult to integrate the yellow fever vaccine into recommended areas.

**CONCLUSIONS:**

Given the ongoing coronavirus disease 2019 pandemic, it is necessary to strengthen measures for the surveillance, prevention, and control of yellow fever with multilateral cooperation between countries.

Brazil is currently experiencing its greatest sylvatic yellow fever (YF) epidemic ever, reaching a record number of cases (2,114 cases) between 2016 and 2018. The epidemic is centered predominantly outside the Amazon region (2,104 cases), and has reached more than 4 times the number of all cases over the previous 20 years. As YF is typically a multimodal epidemic, we consider the period from 2016-2018, after the epidemic began in the Central-West region of Brazil in 2014 and spread into the Southern region, to be part of the two or more waves marking the peak of an epidemic^1^.

This particular epidemic was marked by the persistent transmission of the virus in non-human primates (NHP), even in periods of low seasonality, which has subsequently affected areas previously considered to be without risk, such as the states of Rio de Janeiro and Espírito Santo, and the eastern portions of Bahia and São Paulo. This increase in the territory affected by the virus, along with an associated increase in the recommendation of anti-yellow fever vaccination, has increased vaccine demand by 30,000,000 doses. Currently, vaccination is being expanded to include the whole country. YF transmission has been so intense that there have been reports of cases originating in Brazil documented in France, the Netherlands, Romania, Switzerland, Argentina, and Chile^2^.

In addition to the known epidemic described above, the Evandro Chagas Institute of the Health Surveillance Secretariat of the Brazilian Ministry of Health detected the YF virus in *Aedes albopictus* mosquitoes captured in the state of Minas Gerais. It is widely known that these insects are capable of bridging the gap between sylvatic and urban transmission, increasing the risk of YF re-urbanization^3^ ^,^ ^4^.

In regards to the overlap of this YF epidemic with the ongoing coronavirus disease 2019 (COVID-19) pandemic, the present report aimed to present the strategies adopted by the Pan American Health Organization (PAHO) in order to improve the understanding of various types of international technical cooperation in health care. The present report entails an integrative review of the cooperative actions carried out by PAHO in coping with the YF epidemic from 2016 to 2020.

The Ministry of Health (MoH), in compliance with the International Health Regulations (IHR), notified PAHO of known YF cases in January 2017. PAHO then issued an epidemiologic alert for all American countries, in accordance with the IHR, which has been found to be an effective instrument with which to start response activities in the event of a disease outbreak.

In Brazil, PAHO has played a central role in the technical handling of the current YF emergency, collaborating with the MoH and MoH-partnered research groups in regards to the national response and preparation of other countries within the Americas that may be affected by this YF epidemic. Through the direct participation of their specialized teams *in situ* and providing of supportive interventions in different response areas, PAHO facilitated the dialogue of several health institutions, and contributed to the knowledge regarding YF transmission, as described in the post-event evaluation and control actions of YF in the state of Minas Gerais^5^.

The response to the ongoing YF epidemic has brought together the expertise of scientists from different disciplinary fields, involving clinical, epidemiological, virological, laboratory, and public policy aspects, forming a multidisciplinary and multi-institutional team. It is worth mentioning that the established research capacity in Brazil was decisive in ensuring a fast and accurate response. The participation of the Evandro Chagas Institute and the Oswaldo Cruz Foundation (Fiocruz), which are federal institutes whose missions are to produce, disseminate, and share knowledge and technology that contributes to bettering the health and the quality of life of the Brazilian population, was very important to the YF response. In addition to generating knowledge, Fiocruz is also responsible for producing immunobiological and diagnostic tests.

Historically, technical cooperation in the fight against YF was began with the inception of PAHO, which has addressed the disease since its creation in 1902, as the International Sanitary Bureau of the American Republics. The program was institutionalized at the first International Sanitary Convention of the American Republics in 1902, during which the vector-borne transmission of YF, a historically neglected theme, was discussed. PAHO is proud to have achieved the elimination of urban YF with the eradication of *Aedes aegypti* in 1942. 

Honoring its mission “to promote equity in health, to combat disease, and to improve the quality of, and lengthen the lives of the peoples of the Americas”, PAHO has spared no efforts to tackle this epidemic, which has been supported by the Global Outbreak Alert and Response Network (GOARN).

PAHO's technical cooperation occurred in several ways: training and workshops to exchange experiences; carrying out technical cooperation in the areas of patient management and epidemiological, entomological, laboratory, and epizootic disease surveillance; and organization of an assistance network. Equally important was the dissemination of knowledge with support for the creation of distance education courses, the publication of handbooks, the hiring of personnel for laboratory and field epidemiology activities, and the acquisition of strategic items, including dry ice, syringes, software licenses, vaccines, and supplies for molecular and histopathological diagnosis.

PAHO also organized, for the first time on the American continent, a meeting for the Eliminate Yellow Fever Epidemics (EYE) strategy, including the World Health Organization (WHO), United Nations International Children’s Emergency Fund (UNICEF), and Global Alliance for Vaccines and Immunization (GAVI), with more than 130 partners. PAHO’s technical cooperation supported the decision of the MoH to adopt a single-dose vaccine, which had been recommended by the organization since 2013, and the use of fractional doses to support the vaccination needs of more than 39,000,000 people. The 7,648,772 fractional doses used provided savings of more than 6,000,000 doses. Figure 1 shows a timeline of the primary milestones of cooperation in facing YF.

**FIGURE 1:**
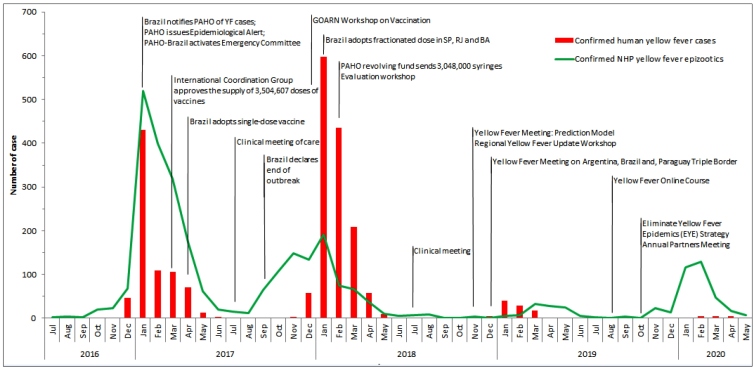
Confirmed yellow fever cases in humans and non-human primates by month, and timeline of PAHO cooperation in responding to the 2016-2019 sylvatic yellow fever epidemic in Brazil.

Professionals hired by PAHO, who then transferred to Brazilian National Laboratories, had significant findings of the YF virus in monkeys of the Callitrichidae family. Managerial support for decisions to expand the recommended vaccination area, adjust the vaccination schedule (adoption of a single dose), and adopt a campaign for fractional doses are also among the cooperative activities carried out by PAHO in Brazil during the current YF epidemic^6^ ^,^ ^7^. 

It is worth emphasizing PAHO's participation in the genomic surveillance of YF, a decisive measure to direct Brazil’s response and reaction to national and international concerns about transmission dynamics, including the type of transmission cycle (sylvatic or urban), virus travel speed and origin, and level of introduction of the virus (initial, single, or reintroduction), etc. In this regard, it is also necessary to mention PAHO's support of the studies that culminated in the publications “Genomic and epidemiological monitoring of yellow fever virus transmission potential” and “Yellow fever virus reemergence and spread in Southeast Brazil, 2016-2019”^8^ ^,^ ^9^.

The present technical report is necessary due to the weakening role of the World Health Organization (WHO) in the coordination of efforts against COVID-19, and the non-compliance of various nations to the IHR. These factors, along with the reduction of funding to WHO by powerful nations, have diminished both the spirit of solidarity between nations and the multilateral approach needed to respond adequately to the pandemic^10^.

The ongoing YF epidemic in Brazil continues to show a significant rate of transmission in the Central-West and Southern regions of Brazil, continuing towards Paraguay and Argentina. The COVID-19 pandemic has contributed to the failure of Brazil to meet the YF vaccination coverage goals, and has made it difficult to implement the YF vaccine in recommended areas. During the ongoing COVID-19 pandemic, it is necessary to strengthen measures for the surveillance, prevention, and control of YF, and multilateral cooperation between countries.
